# A novel technique for H-shaped reinforcement of double-barreled aorta in surgical treatment of chronic aortic dissection

**DOI:** 10.1016/j.jvscit.2024.101543

**Published:** 2024-05-23

**Authors:** Shun-Ichiro Sakamoto, Atsushi Hiromoto, Kenji Suzuki, Takayoshi Matsuyama, Shin-ichi Osaka, Yosuke Ishii

**Affiliations:** a Department of Cardiovascular Surgery, Nippon Medical School Musashikosugi Hospital, Kawasaki-shi Kanagawa, Japan; b Department of Vascular Surgery, Shioda Hospital, Chiba, Japan; c Department of Cardiovascular Surgery, Nippon Medical School, Tokyo, Japan

**Keywords:** Chronic aortic dissection, Double-barreled aorta, Fenestration, Reinforcement

## Abstract

Anastomosis of the prosthetic graft to the double-barreled aorta with intimal flap fenestration is a useful technique in surgery for chronic aortic dissection. Conversely, anastomosis to the false lumen's outer wall is prone to complications such as pseudoaneurysms, but little is known about the technique of reinforcing the double-barreled aorta. In this report, we describe a surgical case of chronic aortic dissection in which an H-shaped prosthetic graft was sutured to both aortic lumens, including the intimal flap, to prevent complications at the anastomosis site.

In the treatment of chronic type B aortic dissection, false lumen closure can potentially impair renal, visceral, and spinal flows in patients with arteries supplying the organs originating from the false lumen. Open surgery for this aortic pathology involves prosthetic graft replacement with preserved blood flow of double lumens.^1^^,^^2^ In this procedure, the aortic wall on the false lumen side is fragile and consists of a single layer of the adventitia, which may result in a pseudoaneurysm at the anastomotic site. In this report, we describe a unique method of using an H-shaped trimmed prosthetic graft to reinforce the intimal flap and aortic wall in the double aorta of chronic aortic dissection.

Written informed consent for publication of the patient's details and images was obtained from the patient before drafting the manuscript.

## Case report

The patient was a 63-year-old man. He underwent total arch replacement in 2004 for Stanford type A, DeBakey type I acute aortic dissection and ascending replacement for a pseudoaneurysm at the proximal anastomosis in 2007. Thereafter, he was treated for hypertension by his primary care physician. In 2022, he was referred to our hospital after chest computed tomography (CT) scan revealed an enlarged distal arch aortic aneurysm.

**Fig 1:**
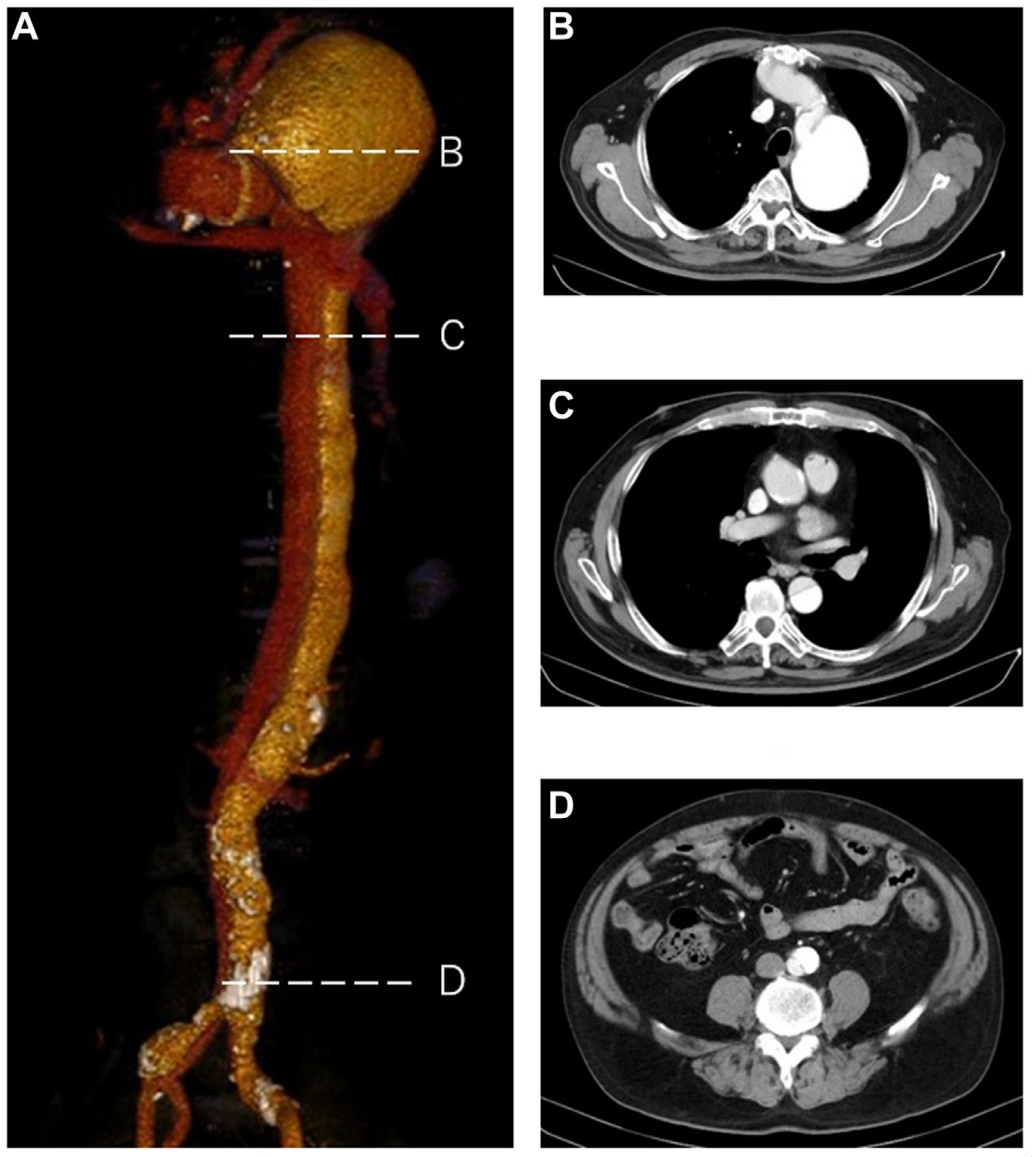
Preoperative contrast computed tomography (CT) scan. **(A)** A three-dimensional (3D) reconstruction of a preoperative computed tomographic image showing an aortic aneurysm of the distal aortic arch associated with a patent false lumen owing to the intimal tear adjunctive to the elephant trunk. A horizontal view matching the dotted line and the letter is shown on the right. **(B)** A diameter of 7.4 cm maximum dilatation of the false lumen in the distal aortic arch. **(C)** Double-barreled thoracic descending aorta. **(D)** Reentry in the terminal aorta.

Contrast-enhanced CT scan showed that the elephant trunk had descended into the true lumen of the distal arch at the previous surgery, but a false lumen was contrasted from a nearby tear, forming a saccular-shaped aneurysm with a maximum short diameter of 72 mm that compressed the true lumen. A double-lumen chronic aortic dissection of the descending aorta to the common iliac artery was observed, and the intercostal arteries, left renal artery, inferior mesenteric artery, and left common iliac artery were perfused through the false lumen. The second tear was also identified at the terminal aorta level (Fig 1). Open repair was selected based on the risk of false lumen occlusion by thoracic endovascular aortic repair and the associated organ ischemia and paraplegia, as well as the patient's desire to avoid reintervention.

**Fig 2:**
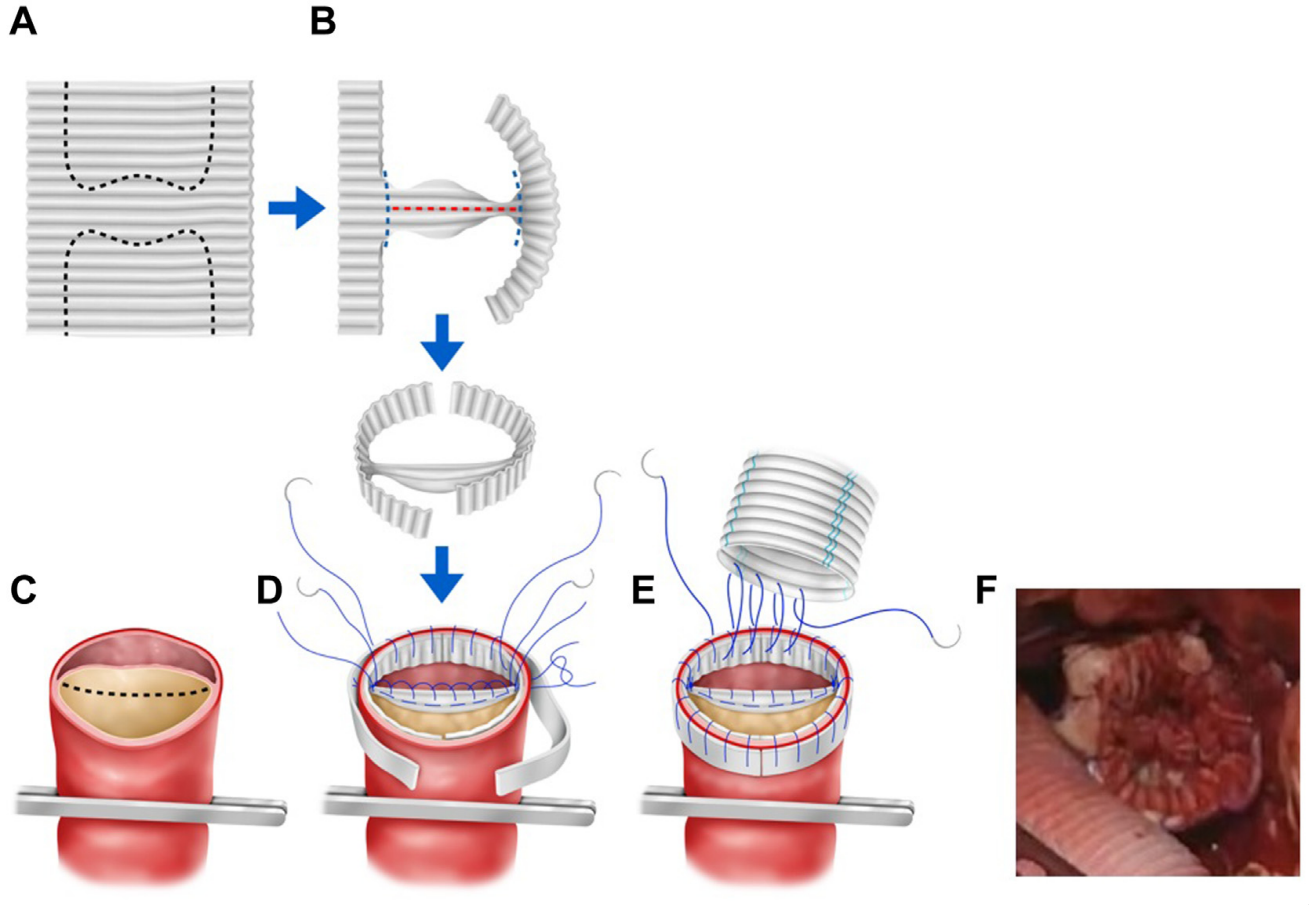
H-shaped reinforcement in the double-barreled aorta. **(A)** H-shaped markings (black dotted line) are made on the sheet removed from the prosthetic graft; the longitudinal diameter of the H-shaped mark corresponds to the aortic circumference. **(B)** The H-shaped trimmed sheet is designed to be mountain folded with red-dotted lines and valley folded with blue dotted lines. **(C)** The intimal flap in the double-barreled aorta is fenestrated by the black-dotted line. **(D)** The folded portion of the sheet is sutured to the intimal flap in two layers with 4-0 polypropylene. The aortic walls of both the true and false lumens are circumferentially sutured with 4-0 polypropylene by placing a sheet on the inside and a felt strip on the outside of the aortic wall. **(E)** The prosthetic graft is anastomosed to the reinforced double-barreled aorta. (**F**) Intraoperative photograph of double-barreled aorta after H-shaped reinforcement.

**Fig 3:**
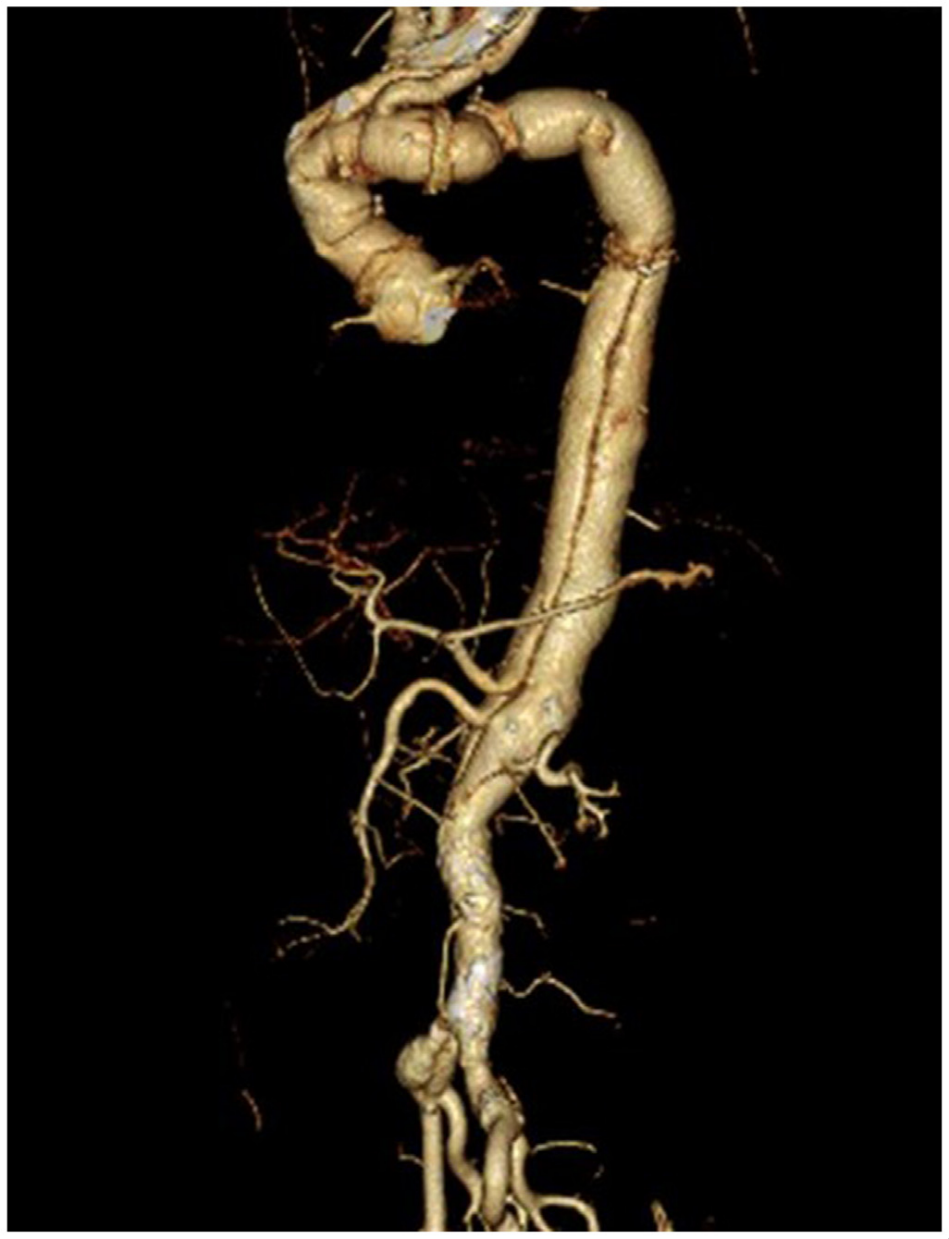
Postoperative contrast computed tomography (*CT*) scan. A three-dimensional (3D) reconstruction of a preoperative computed tomographic image at the 1-year follow-up showing good perfusion of both lumens. No aneurysmal dilatation in the aorta peripheral to the double-barreled anastomosis is observed.

The chest was opened at the left fourth intercostal space, and the aorta was taped distal to the origin of the left subclavian artery. After establishing extracorporeal circulation in the right femoral artery and vein, the aorta was clamped. The arch aortic aneurysm was incised on the beating heart and a 24-mm prosthetic graft (J-Graft; Japan Lifeline, Tokyo, Japan) was anastomosed to the elephant trunk. The descending aorta was resected 4 cm above the aortic clamp site, from which the intimal flap was resected caudally in a 10-mm curvilinear fashion. The inner side of the transected aorta, including the intimal flap, was covered with an H-shaped prosthetic graft, and the outer side was covered with felt strips. Polypropylene sutures (4-0) were used to suture and fix both sides to maintain the double-barreled structure (Fig 2). After end-to-end anastomosis of the graft to the double-barreled aorta, the aortic clamp was released, and extracorporeal circulation was terminated. The total operative time was 344 minutes. The patient was extubated on postoperative day 2 and discharged without complications on postoperative day 20. Postoperative contrast-enhanced CT scan at the 1-year follow-up showed that the aorta was double-barrel from just below the anastomosis with good blood flow in both lumens (Fig 3). No pseudoaneurysm or enlargement of the aorta was observed.

## Discussion

Conventional surgical repair for chronic aortic dissection excludes the intimal tear with graft replacement of the aneurysmal portion. There are two types of peripheral anastomoses in this procedure: anastomosis to the true lumen and anastomosis to double-barreled aorta. Distal anastomosis to the true causes thrombosis of the false space and prevents peripheral aortic enlargement. The major problem is that postoperative paraplegia owing to spinal cord ischemia has been observed in 5% of cases.^3^ Organ ischemia is also a concern when major visceral arteries are perfused through the false lumen. Although several reentry tears generally exist around the celiac axis, ischemia is unlikely to occur^4^ when reentry can be confirmed only at the terminal aorta level; as in this case, double-barreled anastomosis or anastomosis to the single lumen with surgical or catheter fenestration may be used to prevent thrombus occlusion owing to blood flow retardation.^5^ However, the latter may cause aortic dilatation owing to increased pressure in the proximal blind end of the false lumen.

Prosthetic graft anastomosis to a double-barreled aorta requires intimal flap fenestration and suturing to the single layer of the adventitia on the false lumen side. The case of a patient who underwent a similar suture technique to treat acute aortic dissection has been reported; however, the suture failed during the chronic phase, resulting in a pseudoaneurysm.^6^ Several patients also underwent false lumen wall incision and intimal flap removal during surgery for chronic dissection, resulting in a pseudoaneurysm at the false lumen suture.^1^ These cases indicate that false lumen wall reinforcement is necessary during anastomosis even in chronic aortic dissection.

Reinforcement of the double-barrel layer has classically been performed with Teflon felt strips in the false lumen wall.^7^ However, little is known about the details of the technique and whether it is used in all cases. Shimamura and Maisawa^8^ described a method of suturing a rectangular or triangular incised intimal flap to the adventitia with additional reinforcement by securing felt strips inside and outside all around the suture line.

The novelty of our reinforcement method is that the aortic luminal side, including the fenestrated intimal flap, is reinforced with a single prosthetic graft patch, which we expect will distribute the blood pressure and suture tension on the flap and adventitia and maintain their strength. A case report of a patient who required flap fixation owing to obstruction of the true lumen by a mobile intimal flap after a double-lumen anastomosis during the surgery for chronic aortic dissection has been described.^9^ A study of intimal flap characterization in chronic aortic dissection reported that more than half of patients showed high intimal flap mobility,^10^ suggesting that reinforcement of not only the false lumen wall, but also the intima is necessary.

Another feature of our reinforcement is its ability to maintain sufficient blood flow of both lumens by maintaining the caliber of both lumens sharing the intimal flap. The false lumen does not always remain open on the distal side of chronic aortic dissection after a double-barrel anastomosis.^11^^,^^12^ In addition, thrombosis and enlargement of the false lumen just below the anastomosis have been reported, even when blood flow through the false lumen is maintained, suggesting that the false lumen diameter at the fenestration site may be narrowed and forward flow may be inadequate.^12^ Although our technique may secure false lumen blood flow, aortic remodeling, such as aneurysm formation, may occur over time, so continual observation is required.

## Disclosures

None.

## References

[1] Roselli E.E., Sepulveda E., Pujara A.C., Idrees J., Nowicki E. (2011). Distal landing zone open fenestration facilitates endovascular elephant trunk completion and false lumen thrombosis. Ann Thorac Surg.

[2] Urbanski P.P., Bougioukakis P., Deja M.A. (2016). Open aortic arch surgery in chronic dissection with visceral arteries originating from different lumens. Eur J Cardio Thorac Surg.

[3] Tian D.H., De Silva R.P., Wang T., Yan T.D. (2014). Open surgical repair for chronic type B aortic dissection: a systematic review. Ann Cardiothorac Surg.

[4] Okita Y., Tagusari O., Minatoya K. (1999). Is distal anastomosis only to the true channel in chronic type B aortic dissection justified?. Ann Thorac Surg.

[5] Pujara A.C., Roselli E.E., Hernandez A.V. (2012). Open repair of chronic distal aortic dissection in the endovascular era: Implications for disease management. J Thorac Cardiovasc Surg.

[6] Saito Y., Tani K., Taniguchi S., Fukuda I. (2017). Endovascular "intimal flap septostomy" for safe landing of a stent graft in an anastomotic pseudoaneurysm of chronic type B aortic dissection. EJVES Short Rep.

[7] Panneton J.M., Teh S.H., Cherry K.J. (2000). Aortic fenestration for acute or chronic aortic dissection: an uncommon but effective procedure. J Vasc Surg.

[8] Shimamura Y., Maisawa K. (2017). Technique to reinforce double-barreled distal aortic anastomosis in the repair of aortic dissection. Kyobu Geka.

[9] Hamaji M., Kono S., Matsuda M. (2008). Repeated true lumen collapse after repair of descending thoracic aneurysm in chronic type B dissection. Gen Thorac Cardiovasc Surg.

[10] Lortz J., Papathanasiou M., Rammos C. (2019). High intimal flap mobility assessed by intravascular ultrasound is associated with better short-term results after TEVAR in chronic aortic dissection. Sci Rep.

[11] Yamana K., Takami Y., Niwa W., Matsuhashi K., Sakurai Y., Amano K. (2023). Mid-term results of distal anastomosis to the true lumen for chronic type B aortic dissection. Heart Vessels.

[12] Goksel O.S., Tireli E., Kalko Y. (2008). Mid-term outcome with surgery for type B aortic dissections: a single center experience. J Card Surg.

